# Reviewing the Literature for Epidemiological Trends of Dengue Disease: Introduction to a Series of Seven National Systematic Literature Reviews

**DOI:** 10.1371/journal.pntd.0003260

**Published:** 2014-11-06

**Authors:** Maïna L'Azou, Jeremy Brett, Grenville Marsh, Elsa Sarti

**Affiliations:** 1 Global Epidemiology Department, Sanofi Pasteur, Lyon, France; 2 Sanofi-aventis Singapore Pte Ltd, Singapore; 3 Scientific and Medical Publications Department, Sanofi Pasteur, Lyon, France; 4 Sanofi Pasteur, Mexico City, Mexico; University of Heidelberg, Germany

There is no licenced vaccine against dengue, but several candidates are in development, one of which is in the final stages of clinical evaluation [1]. Once a vaccine is licensed, having the appropriate surveillance system in the field will contribute to the successful implementation of vaccination programs by providing an accurate picture of the disease epidemiology to help document the impact of vaccination. Gaps in the current data, dengue surveillance, and epidemiological research need to be identified and addressed now. Furthermore, in view of the WHO 2020 targets for dengue to reduce morbidity by 25% and mortality by 50%, a snapshot of dengue epidemiology prior to the introduction of enhanced surveillance practices or the implementation of a vaccine and other dengue control measures is needed [2].

Here we introduce a series of systematic literature reviews, initiated as part of efforts to prepare for the introduction of dengue vaccination programs [3]–[9]. In each of seven countries—Brazil, Colombia, the French territories of the Americas, Malaysia, Mexico, the Philippines, and Thailand—where the disease is endemic, the epidemiological trends of dengue were reviewed and knowledge gaps identified. We sought to understand the known epidemiology in terms of: general indicators (including incidence, attack rate, and seroprevalence), the intensity of epidemics and the frequency of hospitalisation and severe dengue, populations and subgroups at increased risk, the geographical distribution of disease and outbreaks, trends of serotype distribution, and the prevalent surveillance systems and diagnostic capacity.

**Figure 1:**
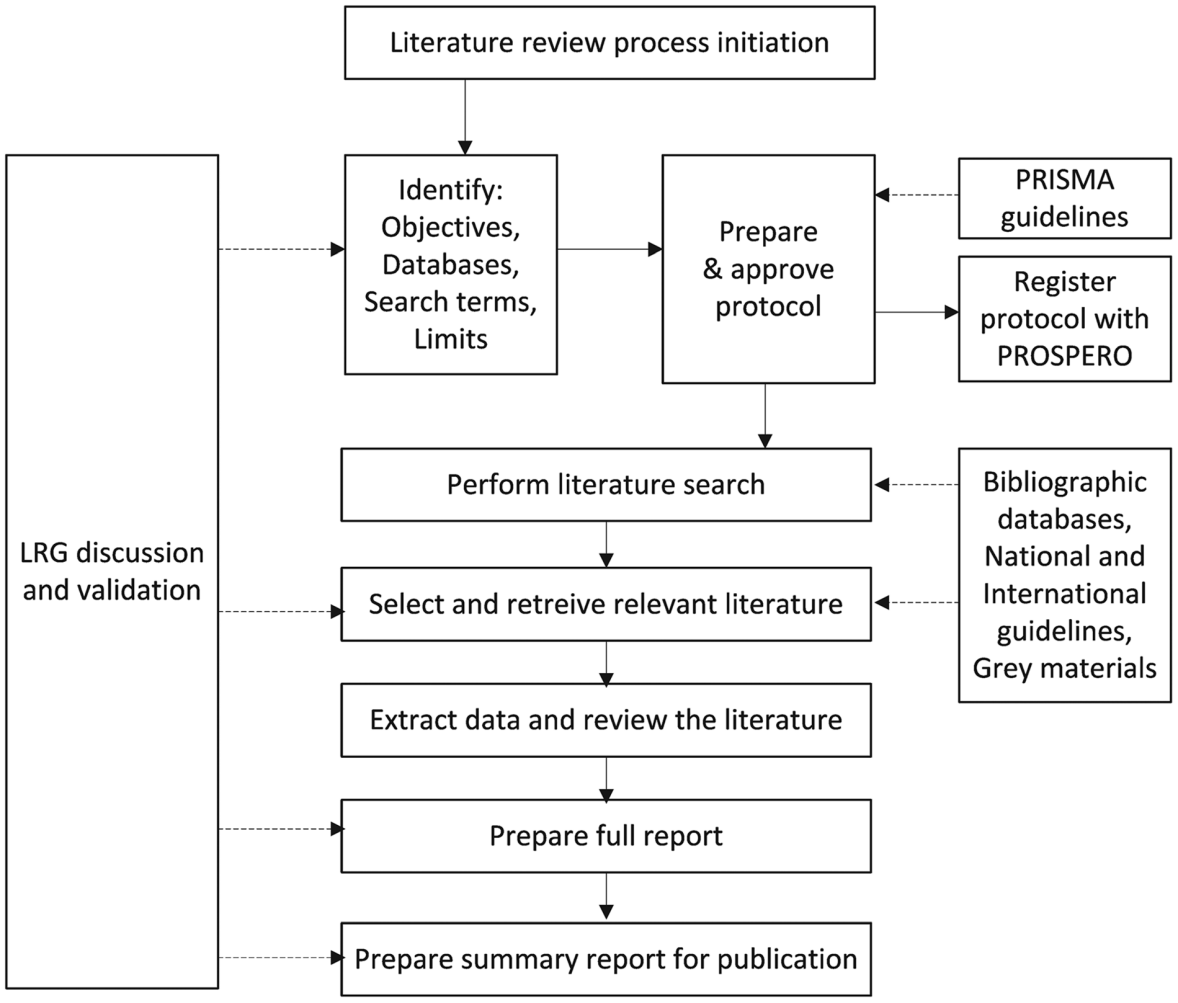
Project organisation and process of the seven national systematic literature review projects. Solid arrows illustrate sequential process steps; dashed arrows illustrate inputs into the process from the LRG or external sources.

We implemented seven collaborative literature review groups (LRG) composed of one or more independent experts in dengue from each country, epidemiologists, and medical advisors from the country and region that met during a series of teleconferences over a period of approximately 18 months. Among the seven countries reported, Brazil was considered first, and the work done by the Brazilian LRG, notably the development of the systematic literature review protocol, in consideration of PRISMA guidelines, served as a pilot and model for subsequent countries. For the other six systematic literature reviews, the LRGs adapted the Brazilian protocol by identifying additional or alternative sources of indexed, English and non-English, peer-reviewed literature; national and international aggregated reports on epidemiological trends; national surveillance data in outbreak summaries; and routine surveillance reports. Search strategies were devised for each electronic database to be reviewed, with reference to the expanded Medical Subject Headings (MeSH) thesaurus, broadly encompassing the terms “dengue”, “epidemiology”, and the “Country”. Databases included MEDLINE (United States National Library of Medicine), Embase (Excerpta Medica), WHOLIS (World Health Organization Library Database), as well as regional databases such as Index Medicus for Southeast Asia Region (WHO Southeast Asia Regional Office), and the SciELO database of articles from national scientific journals from the Latin American region. Key infectious disease, tropical medicine, and paediatric conference papers and posters and grey literature were sought through general Internet searches to complement data gathered in the literature and from searches of organisations. After protocol registration with the PROSPERO international prospective register of systematic reviews, the LRGs reviewed the search results to select articles for further evaluation of the abstract, then full text, including translation when necessary, and selected the literature for analysis. Selected literature was collated and summarized using a data extraction instrument developed as a series of spreadsheets to facilitate analysis and reporting (Figure 1). The LRGs were provided with operational support throughout the process to run the agreed bibliographic searches, retrieve the selected references and extract the data, and to prepare the reports.

An important part of the protocol was the definition of the period for the search. Our objective was not to quantify dengue in absolute terms, nor to provide a complete history of dengue in each country but rather to describe the recent evolution of the disease. Consequently, and given the 3- to 5-year periodicity of dengue outbreaks, we selected a time period of not less than 10 years to allow for the assessment of the evolution of serotype distribution through several epidemics. To facilitate future comparisons between countries, in each case we chose to start our review period on 1 January 2000 and set the cut-off as the date when we initiated each systematic literature review. We hypothesised that setting the start date as 1 January 2000, as opposed to an earlier date, would limit the bias that any differences in surveillance practices over time would have on the results. Our expectation was that surveillance data based on the passive reporting of clinically suspected dengue would represent a significant proportion of the available data. In light of this, and given our objective as described above, we chose not to exclude any data sources based on an assessment of the quality of data but rather to include all relevant, available data.

This series of systematic literature reviews illustrates the unpredictable nature of dengue in terms of the frequency, intensity, and geographic reach of outbreaks, as well as the serotype distribution and number of serotypes circulating in a population at any given time. Differences in the conditions for disease notification between and even within countries also contribute to the observed geographic variability. The disease was seen to affect all age groups, with no clear pattern of population at risk and an age distribution that differed between countries. Highest incidence rates were among adults in Mexico, Malaysia, and in the French Caribbean; adolescents in Brazil and Thailand; and children in the Philippines and Colombia. Furthermore, during the 10–12 year period reviewed, the population most at risk shifted in some countries (in Colombia and Thailand for example), in association with changes in dominant serotype. The unpredictable emergence of new dominating strains resulted in epidemics of varying severity in all countries reviewed. Severity was also seen to vary between locations for the same outbreak. One observed trend over the review period was an increase in the number of hospitalised cases, accompanied by a case fatality rate that decreased to low levels. However, it is important to note that changes to the surveillance system such as the inclusion of virological surveillance, and notably changes to the case definitions used for reporting, confound our understanding of the situation.

In contrast to epidemiological studies that provide detailed and accurate data in a unique spatiotemporal context, surveillance systems are sustainable and provide a broad understanding of the epidemiology over time, enabling, for example, outbreak disease control measures to be implemented. These systems are national and tailored to the specific national situation, and as such, direct comparisons between countries are difficult. This series of systematic literature reviews highlights some common areas where existing systems might be improved upon, including a better understanding of the extent of underreporting, improved specificity and representativeness (for example by the inclusion of the private sector), and the addition of serotype surveillance. Finally, a number of areas for further research were found to be common to several of the countries reviewed. In particular, data on age-stratified seroprevalence, the extent of primary versus secondary infections, serotype and genotype data, and the burden of non-hospitalised cases were lacking. Our understanding of dengue epidemiology would also be improved by a better understanding of the risk factors for severe disease and the role of asymptomatic infections in disease transmission.
